# Lipid metabolism regulator human hydroxysteroid dehydrogenase‐like 2 (HSDL2) modulates cervical cancer cell proliferation and metastasis

**DOI:** 10.1111/jcmm.16461

**Published:** 2021-03-18

**Authors:** Yang Yang, Anna Han, Xinyue Wang, Xianglin Yin, Minghua Cui, Zhenhua Lin

**Affiliations:** ^1^ Department of Pathology and Cancer Research Center Yanbian University Medical College Yanji China; ^2^ Key Laboratory of the Science and Technology Department of Jilin Province Yanji China

**Keywords:** cervical cancer, EMT, HSDL2, lipid metabolism, prognosis

## Abstract

Human hydroxysteroid dehydrogenase‐like 2 (HSDL2) is a potent regulator in cancers and is also involved in lipid metabolism, but the role of HSDL2 in cervical cancer and whether it regulates the progress of cervical cancer through lipid metabolism remains unclear. In this study, we found that the overexpression of HSDL2 was in relation with cervical cancer progression including lymph nodes metastasis and recurrence. HSDL2 could serve as a novel marker of early diagnosis in cervical cancer. HSDL2 also gave impetus to tumorigenesis by initiating and promoting proliferation, invasion and migration of cervical cancer cells (Hela, C33A and SiHa) through EMT. Interestingly, we also searched that HSDL2 participated in oncogenesis by regulating lipid metabolism. In sum, our results gave the novel insight of HSDL2 functions which could be the potential for being the biomarker of prognosis and new target of therapy.

## 1 INTRODUCTION

Cervical cancer is one of the most common gynaecological carcinomas that ranks second in mortality of gynaecological oncology in developing countries.^1^ There are approximately 569 847 new cases and 311 365 deaths each year with a trend of younger age patients.^2^, ^3^ Obviously, cervical cancer is critically threatening the health of women as well as life, and the number of patients is still increasing now.^4^ The mostly new data published by WHO classified cervical cancer into two types: HPV‐negative and HPV‐positive cervical cancer. Hence, the HPV infection is not a single factor causing cervical cancer anymore.^5^ Besides, some researchers suggested that other problem such as gene mutation may involve in the disease initiation.^6^, ^7^, ^8^ Nowadays, although the great development has been made in diagnosis and therapy of cervical cancer, there still exist problems such as false‐negative results in diagnosis, operational risks in surgery and side effects of radiotherapy and chemotherapy.^9^, ^10^ Thus, a new biomarker for diagnosis and prognosis for cervical cancer urgently needs to be carried out.

Human hydroxysteroid dehydrogenase‐like 2 (HSDL2) is a member of short‐chain dehydrogenases/reductases (SDRs) super family which was found in peroxisomes and mitochondria.^11^ This gene in human beings is high homologous to mouse, fruit flies and nematodes, suggesting it is a highly conserved gene. Additionally, HSDL2 located at the chromosome 9q32 with full‐length of 3211 bp encodes a ubiquitously expressed enzyme which contains an N‐terminal SDR domain, a C‐terminal sterol carrier protein 2 (SCP2) domain, a peroxisomal targeting signal (ARL) and conserved motifs like NAD(P)^+^ coenzyme binding sites, enzymatic activity sites.^12^ Normally, HSDL2 is highly expressed in the liver, kidney, prostate, testes and ovaries, but researchers also found the high expression level of the HSDL2 in various cancers such as cholangiocarcinoma, ovarian carcinoma, glioma.^13^, ^14^, ^15^ However, the correlation between HSDL2 and cervical cancer remains elusive. Obviously, cervical cancer is critically threatening the health of women as well as life, and the number of patients is still increasing now.

Lipid metabolism, which has been identified as an essential hallmark in a variety of cancers, is known to contribute to tumorigenesis and metastasis by activating cell signalling pathways.^16^ Some studies demonstrated that increased synthesis of fatty acid is a significant feature in occurrence and development in tumour, which causes more cell membrane lipid synthesis, energy production and signal transduction providing ample support for proliferation and metastasis of tumour cells.^17^ Recently, Shang et al^18^, ^19^ observed that the long noncoding RNA LNMICC promotes lymph metastasis in the cervical cancer through reprogramming fatty acid metabolism, and several research studies suggested that HSDL2 might be a key factor of fatty acid regulatory in lipid metabolism. Unfortunately, study on the mechanism of HSDL2 promoting cervical cancer through the lipid metabolism is limited till date.

In this study, we first investigated that HSDL2 was up‐regulated in cervical tumour tissues than in para‐carcinoma tissues. More essentially, we provided evidence that HSDL2 exerts various effects on biological functions in cervical cancer cells and relates to patients' life span. Meanwhile, our team also newly revealed that HSDL2 triggers the tumorigenesis through lipid metabolism in representative cervical cancer cell lines. To conclude, HSDL2 plays a fatal role in a variety of cervical cell functions, which indicated HSDL2 may become a novel target in biotherapy and prognosis of cervical cancer.

## 2 MATERIALS AND METHODS

### 2.1 Patients and tissue sample collection

We randomly selected 119 cervical cancer, 29 CINs, and 45 normal cervix samples from Shanghai Outdo Biotech Co., Ltd. The consents were acquired from all the patients and this study was approved by the Human Ethics committee and the Research Ethics committees of Yanbian University Medical College in China. The age of patients at diagnosis ranged from 29 to 70 years, with an average age of 50 years, while 81 patients with age ≥50 and 38 patients with age<50. In addition, other clinicopathological characteristics, containing pathological stage, tumour size, HPV infection, lymphatic metastasis and distant metastasis were statistically analyzed. Additionally, we used the criteria of the International Federation of Gynecology and Obstetrics (FIGO) to clinically grade all the patients; 90 cases were FIGO stage I‐II and 110 cases were FIGO stage III‐IV. Besides, the overall and disease‐free survival were also analysed using SPSS.

### 2.2 Cell culture

All the cervical cancer cell lines (Hela, C33A and SiHa) were cultured in DMEM medium (Gibco, Gaithersburg, MD, USA) with 10% foetal bovine serum (FBS), 100 units penicillin and 100 mg/mL streptomycin at a 37°C humidified atmosphere including 95% air and 5% CO_2_.

### 2.3 Transfection

We purchased three different HSDL2 siRNA, including si‐HSDL2‐1, si‐HSDL2‐2 and si‐HSDL2‐3, from RIBOBIO (China). Based on the knockdown effect, control siRNA (si‐control), si‐ HSDL2‐2 and si‐ HSDL2‐3 were selected in this study. The sequence of si‐HSDL2‐2 and si‐HSDL2‐3 were 5ʹ‐CTTCTAGGCACAATCTATA‐3ʹ and 5ʹ‐GCAGCAAAGGATGGAGCAA‐3′. Cells were transfected with 30 nmol/L siRNA using Lipofectamine 3000 (Invitrogen) according to the instruction. pCMV6‐HSDL2 overexpression plasmid was acquired from Origene (Rockville, USA). The HSDL2 plasmid and corresponding empty vector were transfected into three kinds of cervical cancer cells using Lipofectamine 3000 reagent (Invitrogen) following the manufacturer's protocol.

### 2.4 Immunohistochemistry (IHC) staining

Firsty, tissue sections were deparaffinized at 65°C and rehydrated, then aimed to avoid endogenous peroxidase activity, and slides were incubated with 3% H_2_O_2_ in methanol. Next, antigen retrieval was performed in 0.01 mol/L sodium citrate buffer (pH 6.0) at 80°C. After rinsing in PBS three times, slides were incubated with anti‐HSDL2 antibody (1:200 dilution) at 4°C overnight. Slides were then blocked with biotinylated secondary antibodies. Subsequently, slides were immunostained with 3,3′‐diaminobenzidine and counterstained with Mayer hematoxylin. The blots were visualized using an OLYMPUS microscope.

### 2.5 Evaluation of IHC staining

The HSDL2 staining of cervical cancer tissue sections was judged by a couple‐scoring system. The system contains two elements: staining intensity and area extent. The intensity of staining was graded as follows: 0 meant no obvious staining; 1 meant weak staining; 2 meant moderate staining; and 3 meant strong staining. The area extent was graded as follows (proportion of positive cells) : none or <5% positive cells: 0; 5%‐25% positive cells: 1; 26%‐50% positive cells: 2; and >50% positive cells: 3. The total scores were divided into low‐ or high‐expression groups via multiplying the intensity score and the ratio of positive cells score, ranging from 0 to 12. The specimens that scored ≥4 were classified as high HSDL2 expression, and specimens that scored <4 were classified as low HSDL2 expression. The expression level of HSDL2 was categorized as low expression (−, +) and high expression (++, +++) based on the final staining index values.

### 2.6 Cell viability assay

The viability of cervical cancer cells was detected using a MTT assay. The cells were reseeded into 96‐well plates maintained in DMEM with 10% FBS, and grown at 37°C in a 5% CO_2_ chamber for 24 hours, 48 hours, 72 hours, 96 hours and 120 hours. Then, MTT solution was added into culture medium and DMSO was utilized to dissolve formazan crystals. After shaking the plate for 10 minutes, the OD value of each cell at 570 nm of absorbance was measured using a Tecan Infnite 200 Pro microplate reader (Tecan, Switzerland).

### 2.7 Colony formation assay

Cervical cancer cells were reseeded in a six‐well plate and incubated at 37°C and 5% CO_2_ for 15 days. After rinsing with cold PBS, the cells were fixed with methanol and stained with Giemsa. Then, a light microscope was used to count the colonies correctly.

### 2.8 EdU assay

To detect the cell proliferation, a Cell‐Light™ EdU Apollo®567 In Vitro Imaging Kit (RiboBio) was utilized according to the manufacturer's protocol after seeding cervical cancer cells in a 96‐well plate for 24 hours. The result was photographed using an OLYMPUS microscope.

### 2.9 Wound healing assay

After seeding cervical cancer cells in a six‐well plate for 24 hours, the monolayer surface was evenly scratched using 200‐μL pipette tips, which left a 1 mm width of scratch. Subsequently, the cells were washed three times with PBS, and the assigned cells were removed and added a serum‐free medium. Then, initial wounding and cell migration in the scratched area were captured using a microscope at 0 hour and 24 hours. The migration distances were accurately measured by Image J software for further analysis.

### 2.10 Cell invasion and migration assay

A transwell chamber assay was performed to detect migratory and invasive abilities of cells. Cervical cancer cells were seeded on the upper chambers with a 8‐μm pore size polycarbonate membrane coated with 80 μL Matrigel, and serum‐free DMEM media was added to it. Then, 20% foetal bovine serum/DMEM media serving as an attractant was added into a bottom chamber. After 24 hours of incubation, cells were fixed with 4% formaldehyde and stained with 1% crystal violet to count the invading cells in 10 random fields using the microscope. Cell migration was investigated using the transwell assay either, the only difference was no need for Matrigel. This experiment was performed three times for accuracy.

### 2.11 Immunofluorescence (IF)

After growing cells on coverslips in a six‐well plate for 24 hours, the cells were fixed with 4% paraformaldehyde and permeabilized with 0.5% TritonX‐100, and then the cells were blocked with 3% albumin bovine V. After rinsing with PBS, the cells were incubated with E‐cadherin and vimentin antibodies for 1 hour at 4°C. Subsequently, the Alexa Fluor 488 Goat Anti‐Rabbit IgG (H + L) were used to incubation. Then, nuclei were stained by the DAPI with Antifade Mounting Medium (Beyotime, Shanghai, China) and photographed using a microscope.

### 2.12 Western blotting for protein analysis

First, the whole protein was extracted by lysing cervical cancer cells with RIPA buffer before measuring the protein concentration via a BCA protein assay kit (A8020‐5, Roche, Basel, Switzerland). The cells were dissolved with SDS‐PAGE loading buffer and transferred into PVDF membranes. Then, the cells were incubated with diluted primary antibodies at 4°C after blocking with 5% skim milk dissolved in TBST for 1 hour. Primary antibodies used include HSDL2 antibody, E‐cadherin antibody, Vimentin antibody, Snail antibody, Slug antibody (Cell Signaling Technology, Boston, USA), MMP‐2 antibody (Affinity, Cincinnati, USA), FASN antibody, ACSL1 antibody, SREBP1 antibody (Proteintech) and GAPDH antibody (CWBIO). Next, goat antimouse IgG conjugated to HRP was applied as secondary antibody for 1 hour at room temperature. The cells were shortly incubated by ECL detection reagent, then detected by Imager.

### 2.13 BODIPY 493/503 staining

Cells were grown on coverslips in a 12‐well plate with DMEM including 10% FBS, and infected with every group. After culturing for 24 hours, oleic acid (OA) was added to cells to promote the lipid synthesis. After another 24 hours incubation, cells were rinsed with PBS followed by fixing with 4% paraformaldehyde. Then, cells were added BODIPY 493/503 Invitrogen and incubated in the dark place. After rinsing with PBS, cells were stained by Hoechst (Sigma) at 37°C for 2 minutes and photographed using an OLYMPUS BX53 microscope.

### 2.14 Triglyceride assay kit

Cell suspension was centrifuged at 200 *g* for 10 minutes, and PBS was used to wash the precipitates twice after discarding supernatant. The precipitates were kept for further treatment through centrifugation for 10 minutes. Lysate (Triton X‐100) was leveraged for the cell disruption. The solutions presented in protocol were mixed up at 96‐well plate and incubated at 37°C for 10 minutes. Optical density (OD) value at 510 nm was measured and the triacylglycerol content was calculated according to the manufacturer's instruction.

### 2.15 Phospholipid assay kit

Cell suspension was centrifuged at 805 *g* for 20 minutes and supernatant was retained. The solutions were added and mixed in a 96‐well plate according to the protocol and warmed at 37°C for 1 hour with the plate sealer. Washing buffer was used to rinse the plate for five times. Colour development was started by adding chromogenic reagent and stopped by elimination agent. The OD value at 450 nm was recorded.

### 2.16 Statistical analysis

Statistical analysis was performed using SPSS 25.0, GraphPad Prism software 8.0 and JMP software. Data were carried out from at least three independent experiments and expressed as the mean ± SD. The correlation of HSDL2 expression and clinicopathological characteristics were determined using Pearson's chi‐square test. Survival analysis was detected using log‐rank tests. We also did the univariate logistic regression analysis and multivariable logistic analysis. Biochemical experiments were performed in triplicate and at least three independent experiments were evaluated. A value of *P* < .05 was considered to be statistically significant.

## 3 RESULTS

### 3.1 HSDL2 overexpression correlates with progression of cervical cancer

**FIGURE 1:**
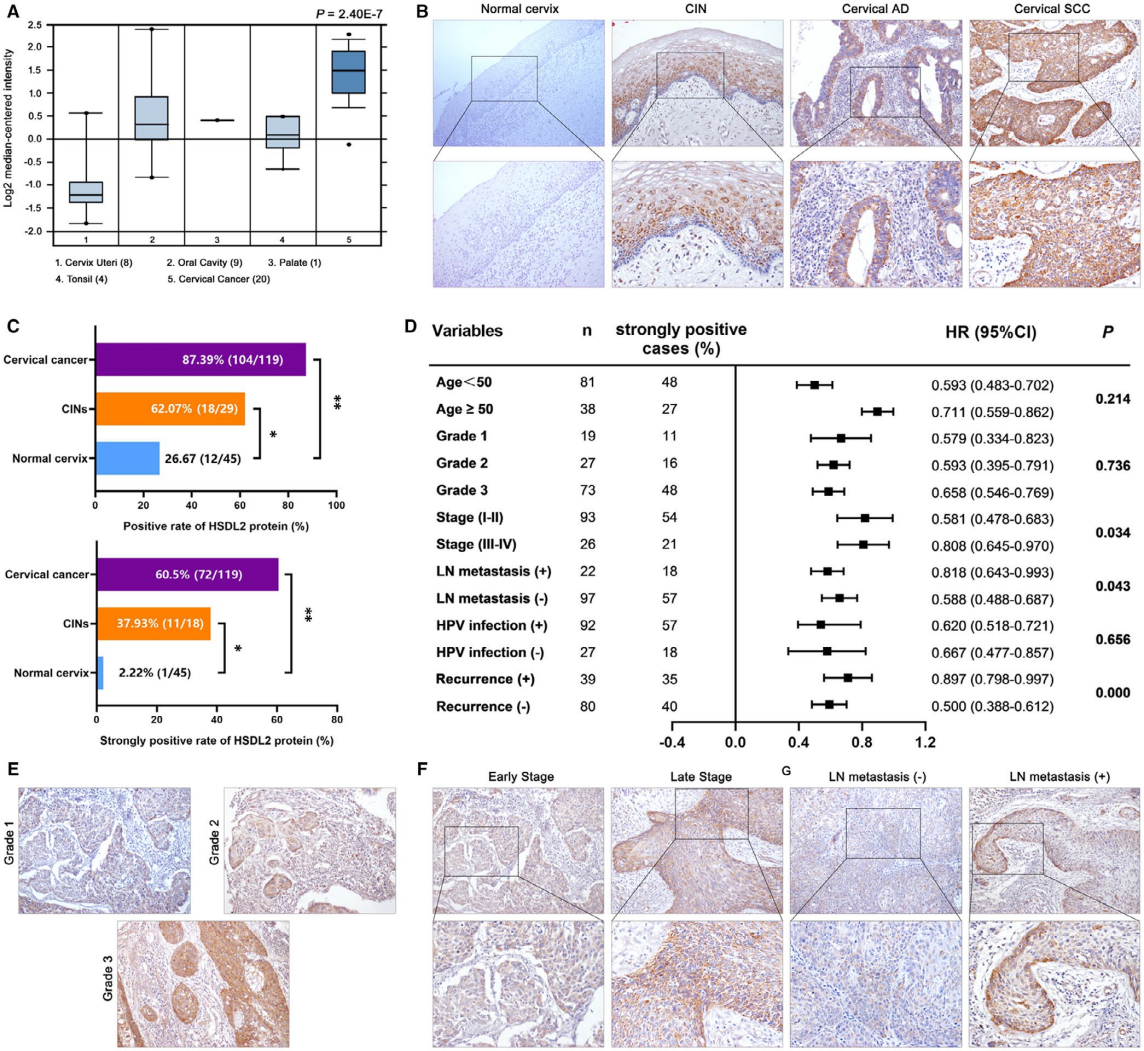
HSDL2 is highly expressed in cervical cancer. (A) Expression level of HSDL2 in normal tissues and cervical cancer samples attained from Oncomine database. (B) Expression level of HSDL2 was investigated by IHC staining in normal cervix, CIN, cervical AD and cervical SCC tissues. Negative staining was showed in normal cervix tissues while moderate staining was presented in CIN and cervical AD samples, and strong staining was exhibited in cervical SCC tissues. (200×, 400×) (C) Statistical results of IHC that HSDL2 protein‐positive and strongly positive staining rates in normal cervix, CIN, cervical cancer tissues. (D) Correlation between HSDL2 expression and the clinicopathological features of cervical cancer. (E) Extremely strong staining of HSDL2 was showed in tissues of grade3 compared to grade1 and grade2. (200×) (F) Staining in tissues with late stage was stronger than samples with early stage. (200×, 400×) (G) HSDL2 was highly expressed in tissues with LN metastasis relative to samples without LN metastasis. (200×, 400×)

Oncomine database presented that the expression of HSDL2 was higher in cervical cancer and lower in non‐cancer tissues (Figure 1A). The IHC assay was performed to investigate the expression of HSDL2 in 119 cervical cancer tissues, 29 CIN and 45 normal cervix tissues. Then, we found negative staining in normal cervix tissues, but strongly and predominantly cytoplasm positive staining in CIN, cervical adenocarcinoma (AD) and cervical squamous cell carcinoma (SCC) (Figure 1B). The positive staining rates and strongly positive staining rates of HSDL2 in cervical cancer tissues are 87.39% (104/119) and 60.5% (72/119), respectively, much higher than in CIN (62.07%, 37.93%) and in normal tissues (26.67%, 2.22%) (*P* < .001, Figure 1C). Subsequently, we observed the association between the expression of HSDL2 and certain clinical parameters. As summarized in forest plot (Figure 1D), HSDL2 overexpression was significantly correlated with stage (*P* = .034), LN metastasis (*P* = .043) and recurrence (*P* = .000), but no significance with age, grade, HPV infection. However, the level of HSDL2 expression was up‐regulated with the rise of grade (Figure 1E), but it had no significance in statistics. Then, we detected the strongly positive staining of HSDL2 in both the samples of late stage (Figure 1F) and lymph node metastasis (Figure 1G), the rates were respectively 61.2% (21/119) and 39.7% (57/119). These observations suggested that HSDL2 might play a critical role in initiation and progression of cervical cancer.

### 3.2 High‐level expression of HSDL2 correlates with poor prognosis

**FIGURE 2:**
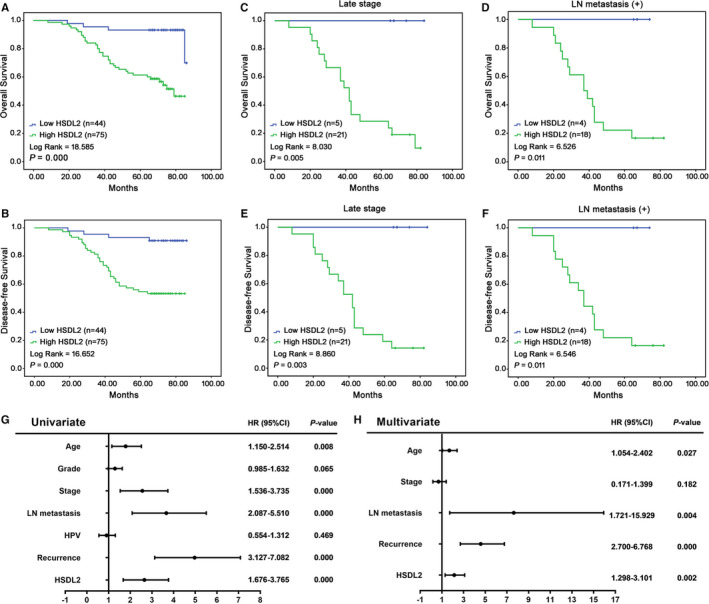
The correlation between HSDL2 expression and prognosis of cervical cancer patients. (A) Overall survival rates of cervical cancer patients in different expression level of HSDL2 analyzed by Kaplan‐Meier. (B) Disease‐free survival rates of cervical cancer patients in different expression level of HSDL2. (C) Overall survival rates of cervical cancer patients with late stage in relation to HSDL2 expression. (D) Overall survival rates of cervical cancer patients with LN metastasis in relation to HSDL2 expression. (E) Disease‐free survival rates of cervical cancer patients with late stage in relation to HSDL2 expression. (F) Disease‐free survival rates of cervical cancer patients with LN metastasis in relation to HSDL2 expression. (G) Forest plots showed the results of univariate and multivariable logistic regression analysis

Next, we further investigated the prognostic value of HSDL2 expression in cervical cancer. We analysed the data of 119 patients using Kaplan‐Meier. The result revealed that the overall survival (Figure 2A) and disease‐free survival (Figure 2B) all correlated with the expression of HSDL2 (*P* = .000, *P* = .000), and the higher level of HSDL2 expression meant poorer prognosis than those lower level of HSDL2 expression. Especially, we detected the significant correlation between survival and HSDL2 expression in the patients with both early and late stage (Figures S1A,B and Figure 2C,D), lymph node metastasis and no metastasis (Figure 2E,F and Figure S1C,D). Forest plots showed the results of univariate logistic regression analysis (Figure 2G). We observed that the high‐level expression of HSDL2 related to stage, LN metastasis and recurrence. Multivariable logistic analysis was followed by above analysis to assess the independent factors (Figure 2H). LN metastasis and recurrence were related independently with high‐level expression of HSDL2. Therefore, we could arrive at the result that HSDL2 may become the considerable prognosis indicator.

### 3.3 si‐HSDL2 inhibits the growth and proliferation capabilities of cervical cancer cells

**FIGURE 3:**
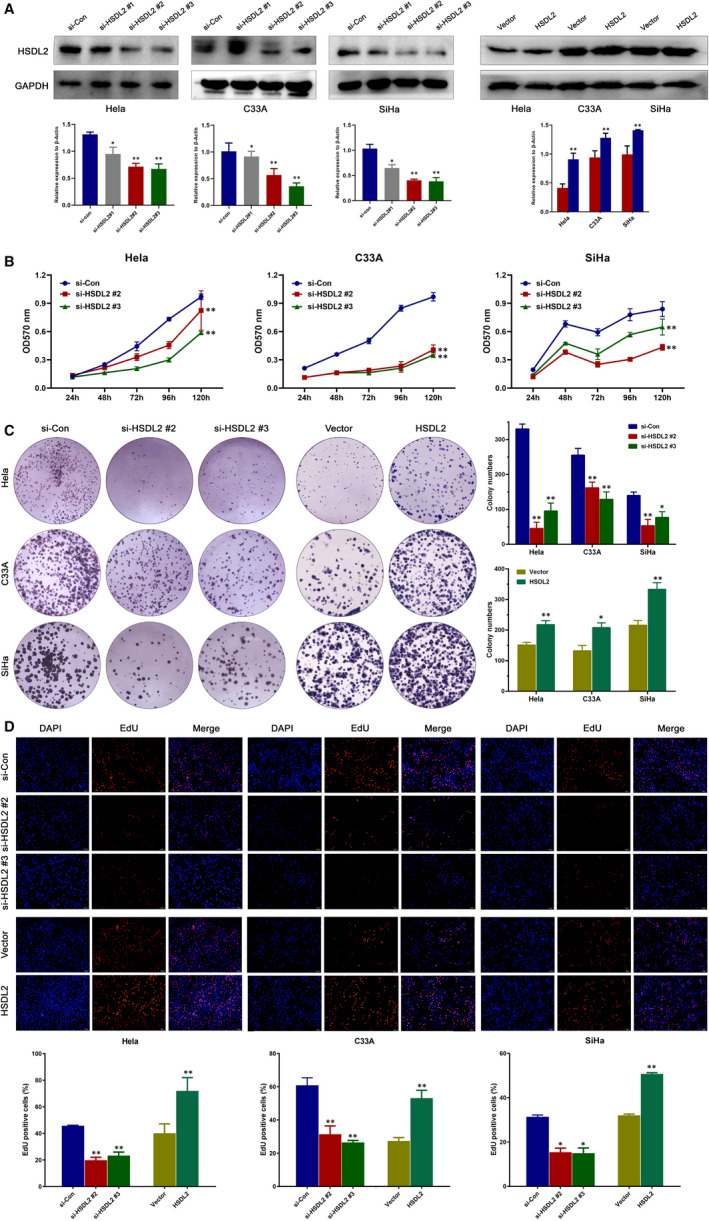
HSDL2 is able to facilitate the progression of cervical cancer. (A) siHSDL2#2 and si‐HSDL2#3 successfully knocked down the HSDL2, whereas HSDL2 plasmid overexpressed the HSDL2 in cell lines containing Hela, C33A and SiHa. (B) The proliferative capacity of cervical cancer cells was obviously attenuated by knockdown of HSDL2 in MTT assay. (C) The colony‐forming ability of cervical cancer cells was reduced by HSDL2 knockdown while enhanced by HSDL2 overexpression. (D) The effect of HSDL2 expression on cervical cancer cell proliferation was detected by EdU assay. *P < .05, **P < .01

To deeply investigate the function role of HSDL2 in cervical cancer cells, we constructed si‐HSDL2 using three different si‐RNAs and simultaneously overexpressed the HSDL2 in cervical cell lines (Hela, C33A and SiHa). Through the Western blotting, we observed that the expression level of HSDL2 was distinctly suppressed in the si‐HSDL2 #2 group and si‐HSDL2 #3 group compared to si‐Con groups (Figure 3A). To determine the function of HSDL2 in growth of cervical cancer cells, the MTT assay was performed and the results indicated that the growth capacity of cervical cells was obviously inhibited in both the si‐HSDL2 groups (Figure 3B). Similarly, based on the colony formation assay, the colony formative ability of cervical cancer cells was also impaired after si‐RNA transfection (Figure 3C). Additionally, thr EdU assay showed that the percentage of EdU‐positive cells markedly decreased in the si‐HSDL2 groups compared to the control groups, which meant that the proliferative ability of cervical cells was reduced in the si‐HSDL2 groups, while the percentage of EdU‐positive cells increased in the HSDL2 groups compared to the vector groups (Figure 3D). To sum up these results, HSDL2 significantly promotes the proliferation of cervical cancer cells.

### 3.4 HSDL2 promotes invasion and migration abilities of cervical cancer through EMT

**FIGURE 4:**
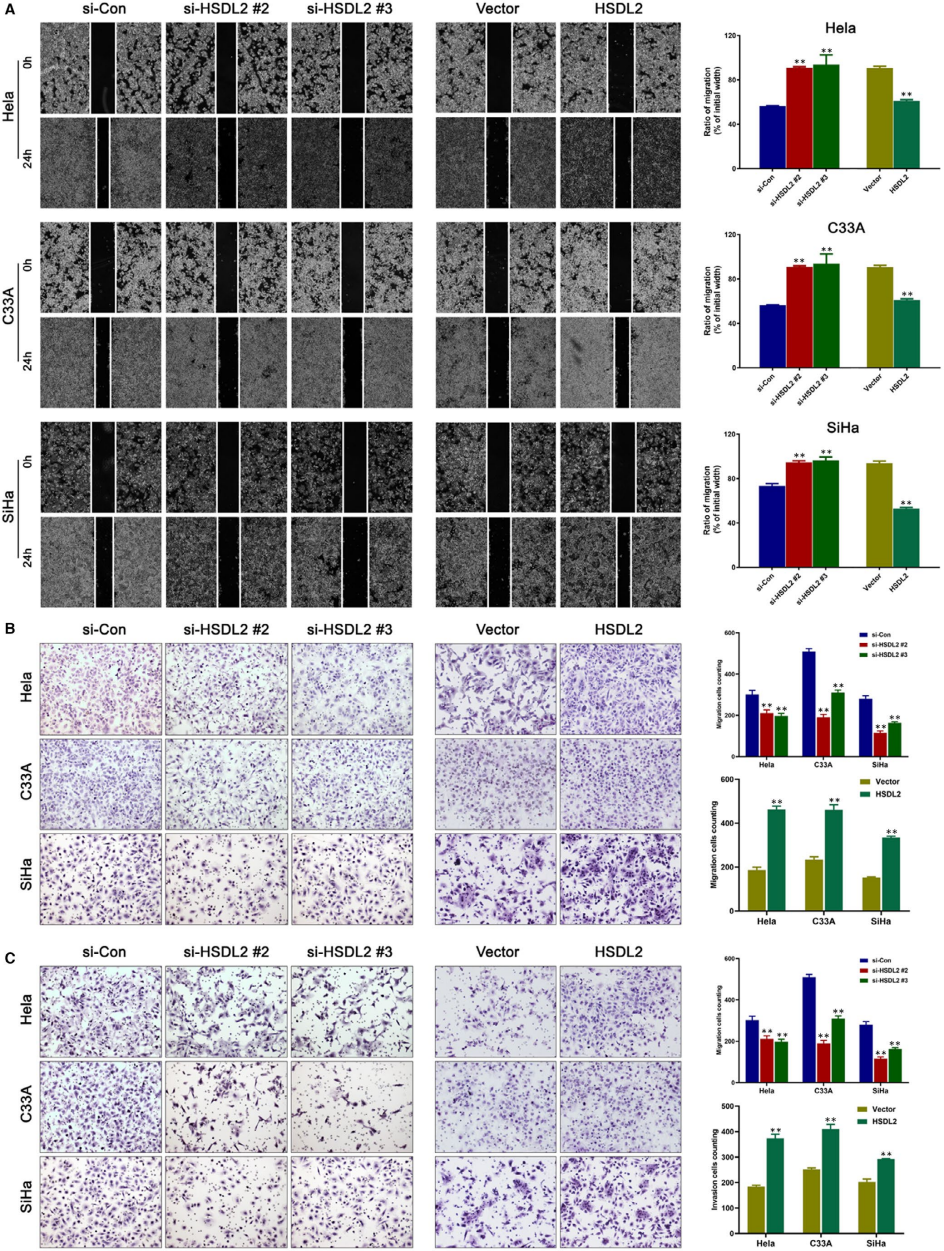
HSDL2 boosts the process of cervical cancer metastasis. (A) The rate of migration into scratched area was slowed in si‐HSDL2 groups and accelerated by HSDL2 overexpression. (B) The effect of HSDL2 expression on migratory ability of cervical cancer cells was determined by migration assay. (C) The invasive capacity was lessened by HSDL2 arrestment and promoted by HSDL2 overexpression. *P < .05, **P < .01

**FIGURE 5:**
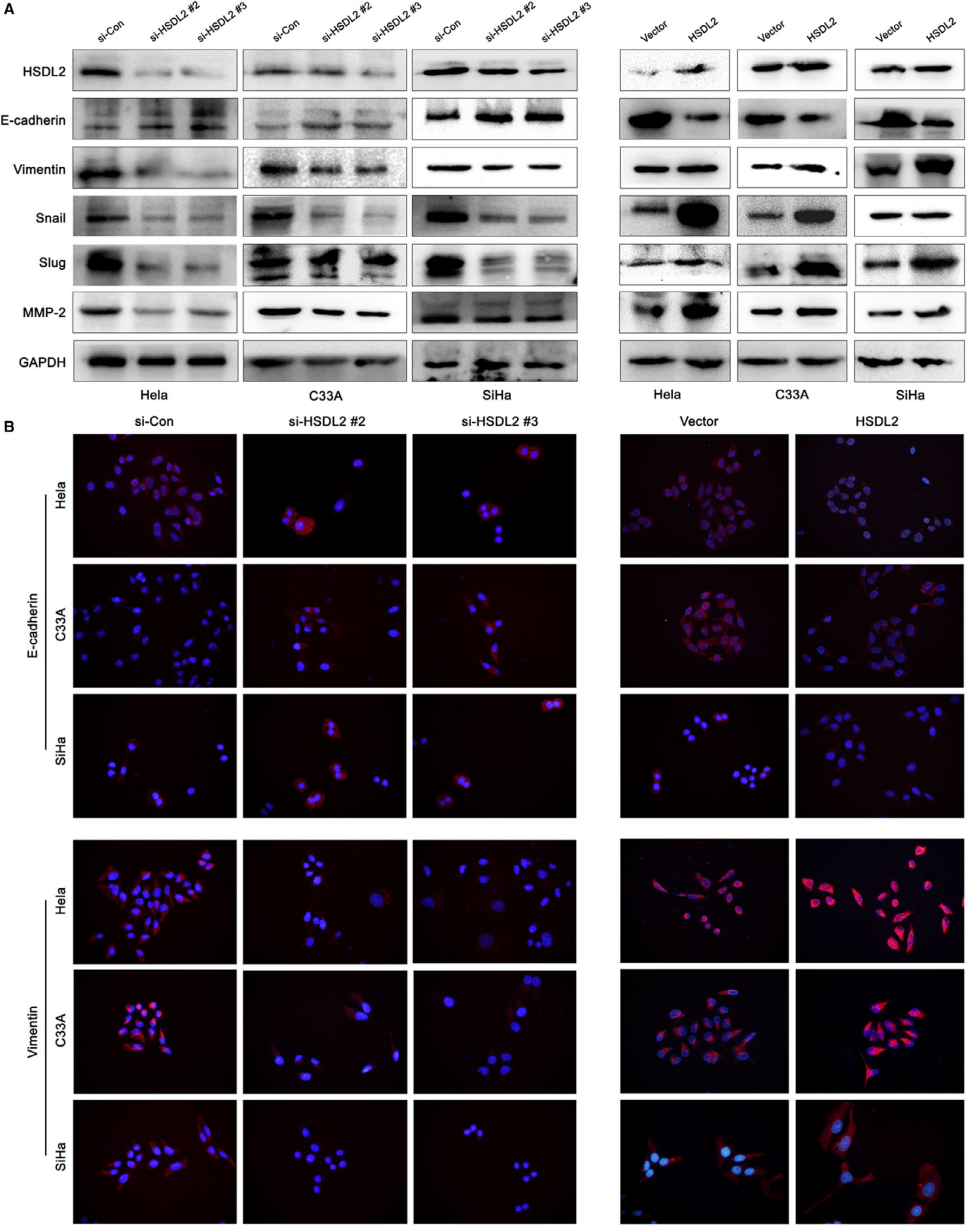
HSDL2 promotes metastasis of cervical cancer through EMT process. (A) Analysis by Western blot assay on EMT markers (E‐cadherin, Vimentin, Snail, Slug, MMP‐2) in Hela, C33A, SiHa cells of both si‐HSDL2 groups and HSDL2 groups. (B) The expression change of E‐cadherin and Vimentin in cervical cancer cells was detected by IF assay. Blue represented cell nucleus and red represented the expression of E‐cadherin and Vimentin in cell cytoplasm

Subsequently, we detected from the wound healing assay that the wound closure area decreased as higher HSDL2 expression (Figure 4A), consistent with it, migration and invasion assay exhibited that the cell migration and invasion abilities of cervical cancer cells obviously declined after HSDL2 knockdown (Figure 4B,C). Based on the information that EMT is the key of cell behaviour and pathologically contributes to the progression of cancers, we applied western blotting assay to determine the correlation between EMT and expression of HSDL2. Compared to the control group, we found that epithelial marker E‐cadherin was up‐regulated in the si‐HSDL2 groups, while mesenchymal markers (Vimentin, Snail, Slug and MMP‐2) were considerably down‐regulated in all three cervical cancer cell lines. Interestingly, HSDL2 overexpression displayed the adverse results (Figure 5A, Figure S2A). Immunofluorescence staining further verified our findings (Figure 5B, Figure S3). These results sufficiently indicated that HSDL2 significantly enhances the ability of invasion and migration of the cervical cancer cells by EMT process.

### 3.5 HSDL2 involves in tumorigenesis by regulating lipid metabolism in representative cervical cancer cell lines

**FIGURE 6:**
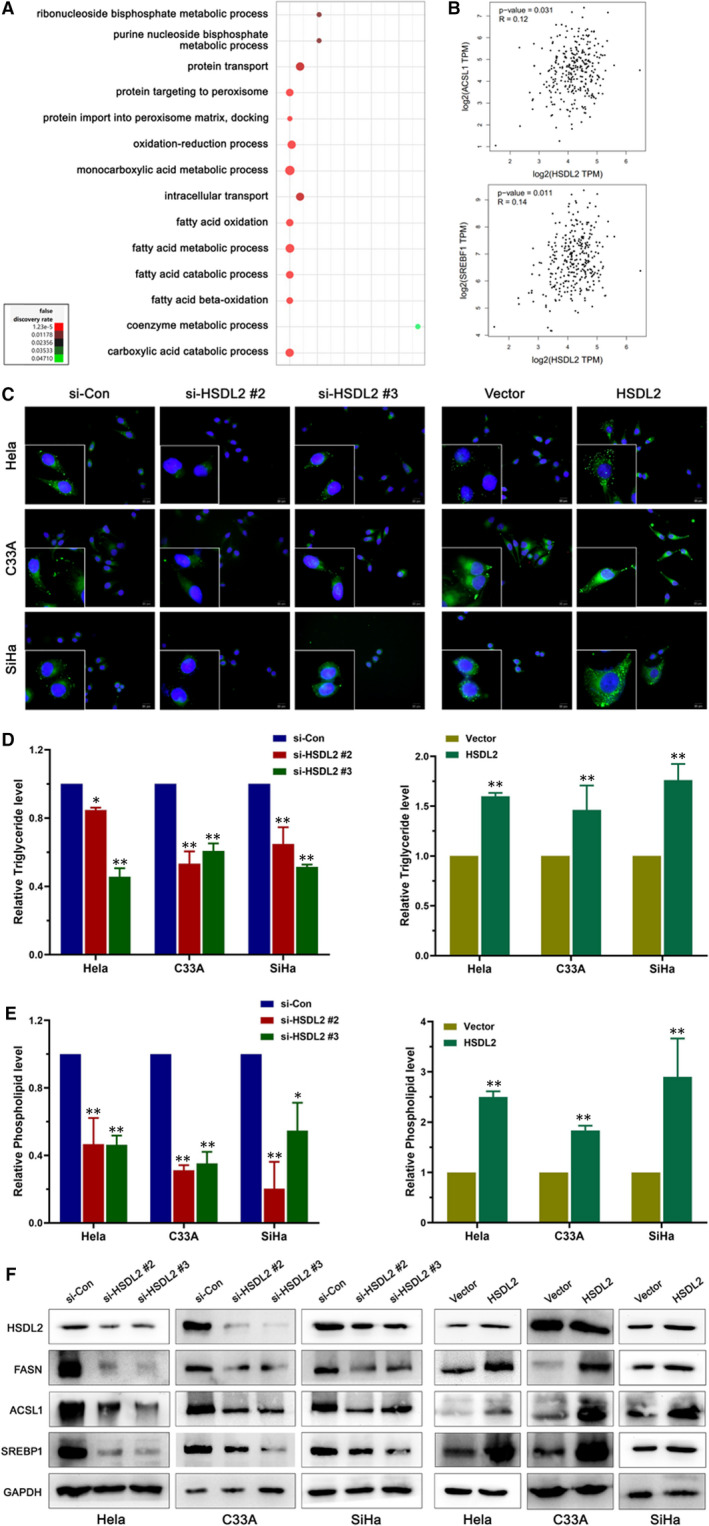
HSDL2 encourages tumorigenesis of cervical cancer through altered FA metabolism. (A) GO analysis indicated fatty acid metabolic process as the most involved GO term in relation to HSDL2 expression. (B) HSDL2 was significantly associated with lipogenesis genes, ACSL1 and SREBF1. (C) HSDL2 knockoff decreased the contents including cholesterol, triglyceride, phospholipid, which could be reversed by HSDL2 overexpression. (D) Correlation between HSDL2 expression and triglyceride level was detected by triglyceride kit assay. (E) Correlation between HSDL2 expression and phospholipid level was detected by phospholipid kit assay. (F) Lipogenesis genes (FASN, ACSL1, SREBP1) were down‐regulated by HSDL2 knockdown which could be inverted by HSDL2 overexpression. *P < .05, **P < .01

Alteration of lipid metabolism has been known as a crucial process in several kinds of cancers.^20^ However, there is no study on whether HSDL2 involves in the reprogramming of cervical cancer lipid metabolism. Therefore, in order to further determine the role of HSDL2 in lipid metabolism, we first filtered the genes with |log2 (fold change) | > 1 by GO analysis; bubble chart showed that HSDL2 closely related to fatty acid metabolic process (Figure 6A). In addition, GEPIA database showed a significant correlation between HSDL2 and key factors of fatty acid synthesis, ACSL1 and SRBEF1 (Figure 6B). These results led us to believe HSDL2 has strong correlation with lipid metabolism in representative cervical cancer cell lines. In this regard, we performed BODIPY 493/503 staining and found out that HSDL2 knockdown reduced the intracellular contents of neutral lipids, while the HSDL2 overexpression had the opposite effect (Figure 6C). In addition, the results of triglyceride and phospholipid assay kits were paralleled with the BODIPY 493/503 staining (Figure 6D,E). Furthermore, we utilized western blotting to probe the effects of HSDL2 on several pivotal lipid metabolic enzymes, including FASN, ACSL1 and SREBP1. The result presented that FASN, ACSL1 and SREBP1 were all fairly down‐regulated in the si‐HSDL2 groups of Hela, C33A and SiHa, which was reversed by the overexpression of HSDL2 (Figure 6F, Figure S2B).

**FIGURE 7:**
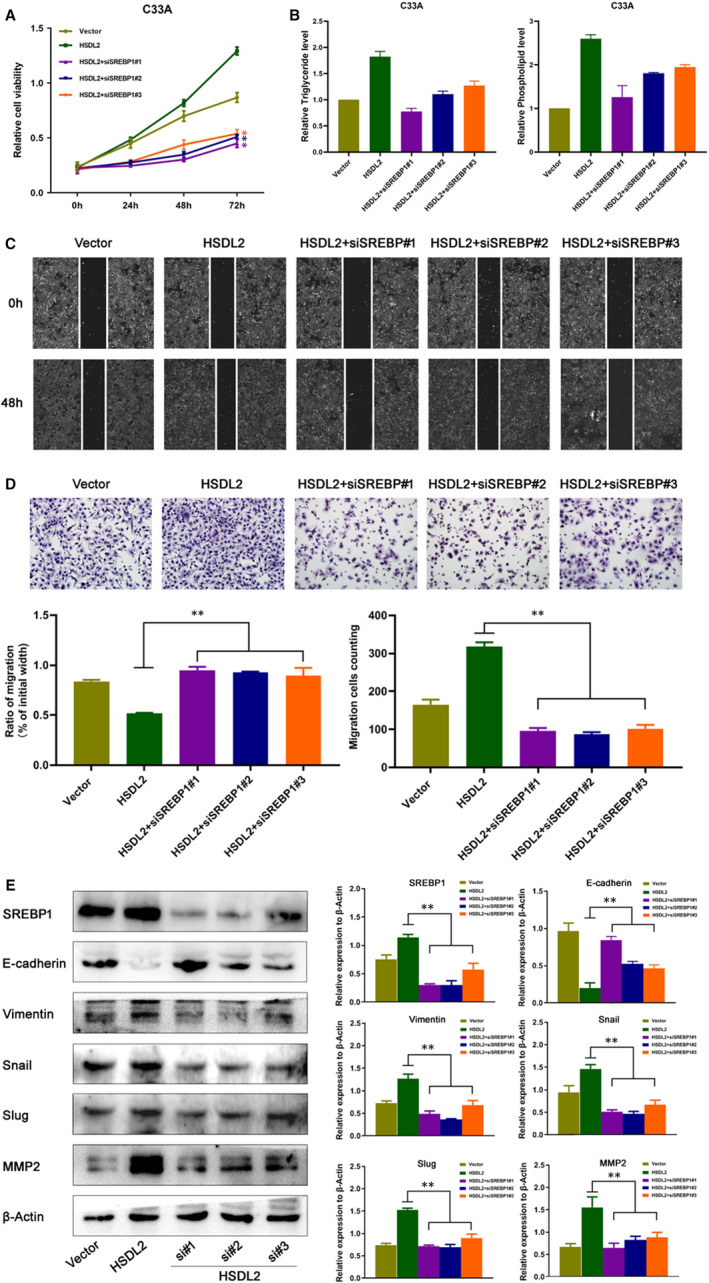
HSDL2 promoted progression of cervical cancer through SREBP1‐mediated lipid metabolism. (A) Representative images showing the MTT assay of cervical cancer cells overexpressing HSDL2 or treated with si‐SREBP1. (B) Triglyceride and phospholipid level were detected by triglyceride and phospholipid kit assay after knockdown SREBP1 in HSDL2 overexpress cells. (C, D) Wound healing (C) and Transwell assay (D) of treated cells. (E) Western blot analysis of EMT markers in treated cells. β‐Actin was used as the loading control. *P < .05, **P < .01

To demonstrate that HSDL2 promoted progression of cervical cancer through lipid metabolism, we knockdown the expression of SREBP1 in HSDL2‐overexpressed cells and found that SREBP1 siRNA eliminated the effects of HSDL2 in inducing cervical cancer cell lipid metabolism, proliferation and abolished HSDL2‐mediated cell migration (Figure 7). Furthermore, the depletion of SREBP1 increased the expression of E‐cadherin and decreased the vimentin, Snail, Slug and MMP2, which reversed the HSDL2‐stimulated mesenchymal cell phenotype. Overall, the above consequences supported the conclusion that HSDL2 may promote progression of cervical cancer through lipid metabolism.

## 4 DISCUSSION

Women in today are concerned fairly about the cervical cancer due to its increasing incidence and high mortality. Although cervical cancer vaccine has been used these days, some researchers suspected that causes of cervical cancer could not just ascribe to HPV infection.^21^, ^22^, ^23^ Thus, it is urgently needed to detect the mechanism of cervical cancer on the molecular level.

HSDL2, a member of SDRs super family, which could be found in peroxisomes and mitochondria, was indicated in growing body of literature about the vital role in the cancer progression.^24^, ^25^ Hence, we speculated that HSDL2 may regulates development of cervical cancer either, and performed a series of assays to demonstrate the deduction. We first examined the strong staining in the species of cervical cancer through IHC, demonstrating that HSDL2 expression significantly correlated with the cervical cancer. Besides, we found the overexpression of HSDL2 could be a determinant of cervical cancer development, such as clinical stage, LN metastasis and recurrence. Of note, HSDL2 overexpression was significantly related to poor prognosis of cervical cancer patients even including patients with early stage and without LN metastasis. These results were in agreement with the study of Sun et al,^13^ which reported that HSDL2 was strongly expressed in ovarian cancer samples and associated with TNM stage, LN metastasis and pathological grade, which could also induce poor prognosis. Additionally, Dong et al^24^ observed that HSDL2 was strongly positive staining in the breast cancer, and detected the correlation between highly expressed HSDL2 and LN metastasis, histological stage and worse prognosis. Therefore, HSDL2 may involves in the initiation and development of cervical cancer, which could become a novel predictor of the cervical cancer.

It is already known that cell proliferation and migration is the first step of cancer process.^26^ A study^27^ suggested recently that bladder cancer cell proliferation and colony formation were evidently attenuated by sh‐HSDL2 lentivirus transfection, then apoptosis of cancer cells would be increased. Hence, we wondered whether the failure of HSDL2 is lethal in the cervical cancer. In this research, we revealed that depletion of HSDL2 arrested the cell growth whereas overexpression of HSDL2 accelerated the rate of cell proliferation. In addition, we found that epithelial marker, E‐cadherin was up‐regulated obviously in the si‐HSDL2 groups, meanwhile, mesenchymal markers such as vimentin, Snail, Slug and MMP‐2 were down‐regulated. This consequence substantially indicated the relevance between HSDL2 and the ability of migration and invasion of cervical cancer cells, and it was agreed again through the wound healing, migration and invasion assay. Broadly speaking, we knew that HSDL2 regulates the proliferation, migration and invasion of cervical cancer cells and might be a new target of molecular therapy of metastatic cervical cancer.

It is well‐documented that lipid metabolism acts as vital one in the metastatic colonization in remote organs due to the fatty acid (FA) would supply the energy to metastatic cancer cells.^28^, ^29^ In the current study, cervical cancer has already been acknowledged as one of the cancers which have involvement with the lipid metabolism. For instance, Shang's team^15^ elucidated that FABP5 induced reprogramming of lipid metabolism in representative cervical cancer cell lines to promote the LN metastasis. Concurrently, Shang and colleagues revealed that BMI ≥ 25 (kg/m^2^) was an independent element of pelvic LN metastasis and poor prognosis in the cervical cancer to further support the aforementioned view. In addition, Jiang et al^29^ investigated that PRRX‐1 induced EMT and further led to the reprogramming of FFA metabolism to promote the metastasis in salivary adenoid cystic carcinoma. Interestingly, HSDL2 locates in peroxisomes and mitochondria which are especially pivotal for the lipid activity. But whether there is a correlation between HSDL2 and lipid metabolism in representative cervical cancer cell lines remains unclear. Hence, our group intended to find the answer about if HSDL2 involves tumour progression by regulating lipid metabolism. GO analysis exhibited that HSDL2 intimately related to the fatty acid process. Accordingly, we observed triglyceride and phospholipid were lessened in the HSDL2‐depleted cervical cancer cells, while the opposite result was shown in the HSDL2‐overexpressed cervical cancer cells. In addition, western blotting experiments were carried out to confirm that HSDL2 plays an important role in fatty acid metabolism. The critical enzymes of lipid metabolism such as FASN, ACSL1 and SREBP1 were all down‐regulated after depletion of HSDL2 and up‐regulated after overexpression of HSDL2. In addition, SREBP1 knockdown clearly repressed proliferation, migration and lipid metabolism by using siRNA in HSDL2‐overexpressing cells, implied that HSDL2 promoted progression of cervical cancer through lipid metabolism. The outcome above reminded us that HSDL2 functions as the momentous factor in altered lipid metabolism promoting cancer progress. It highlights the applicability of HSDL2 as a potential target of the wide range of therapeutic methods. But it is still needed to get insight into the correlation between EMT and lipid metabolism and how they link together to induce metastasis of cervical cancer.

In conclusion, we not only observed the degree of expression of HSDL2 and its association with clinical variables and prognosis, but also unravelled the contribution of HSDL2 with the support of cell‐based assays and the evaluation of key proteins that are involved in the regulation of signalling networks linked to proliferation as well as migration and invasion of cervical cancer cells. Most importantly, the new insights were given into the role of HSDL2 in the lipid metabolism of cervical cancer. All these consequences significantly implied that HSDL2 is a novel but strong potential predictor as well as therapeutic target of cervical cancer, which could bring benefit to cervical cancer patients in the future.

## CONFLICT OF INTEREST

The authors confirm that there are no conflicts of interest.

## AUTHOR CONTRIBUTIONS

**Yang Yang:** Data curation (equal); formal analysis (equal); project administration (equal); resources (equal). **Anna Han:** Data curation (equal); formal analysis (equal); investigation (equal); methodology (equal). **Xinyue Wang:** Methodology (equal); writing‐original draft (equal); writing‐review & editing (equal). **Xianglin Yin:** Writing‐review & editing (equal). **Minghua Cui:** Supervision (equal). **Zhenhua Lin:** Conceptualization (equal); funding acquisition (equal); supervision (equal); visualization (equal).

## Supporting information

Supplementary Material

## Data Availability

The data that support the findings of this study are available from the corresponding author upon reasonable request.
