# Supplementary material for: Lipid metabolism regulator human hydroxysteroid dehydrogenase‐like 2 (HSDL2) modulates cervical cancer cell proliferation and metastasis
Source: J Cell Mol Med. 2021 Mar 18;25(10):4846–59. doi: 10.1111/jcmm.16461 (PMC8107089; doi:10.1111/jcmm.16461)
Supplement: Supplementary file 1 — Supplementary Material [file JCMM-25-4846-s001.docx]

**Supplementary Files:**

**Supplemental Figures:**


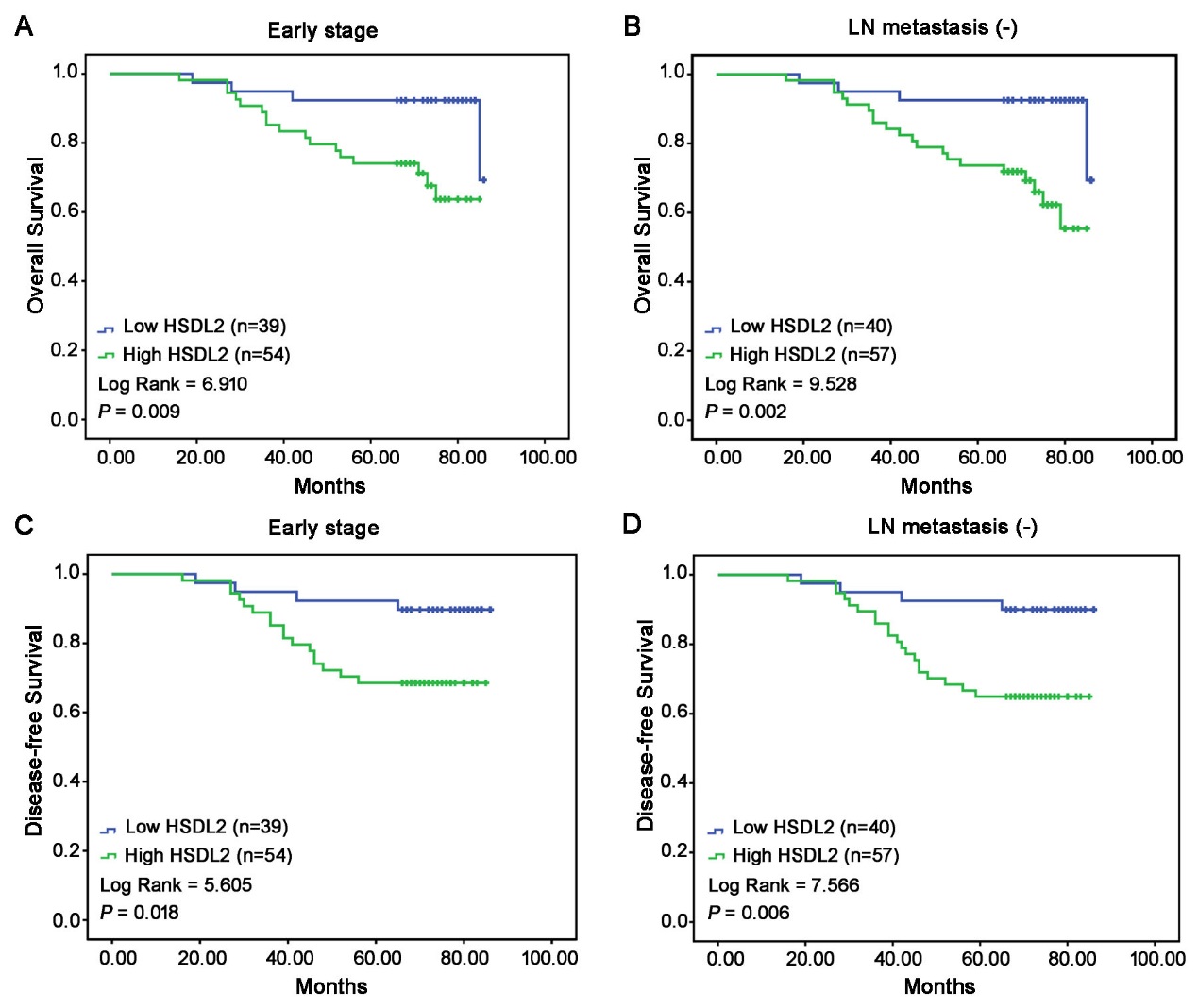


**Supplemental Figure 1:** (A) Overall survival rates of cervical cancer patients with early stage in relation to HSDL2 expression. (B) Overall survival rates of cervical cancer patients without LN metastasis in relation to HSDL2 expression. (C) Disease-free survival rates of cervical cancer patients with early stage in relation to HSDL2 expression. (D) Disease-free survival rates of cervical cancer patients without LN metastasis in relation to HSDL2 expression.


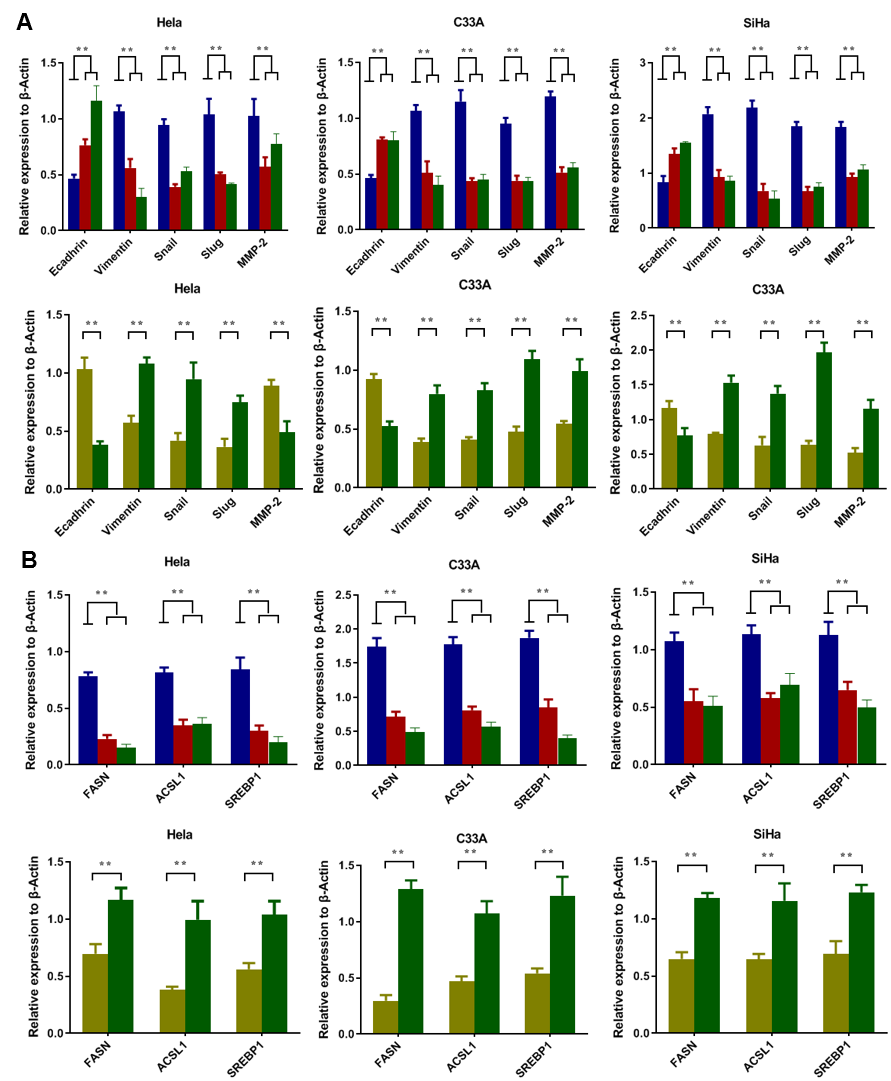


**Supplemental Figure 2: (A)** Statistics of Western blots for Figure 5A. **(A)** Statistics of Western blots for Figure 6F. The data were presented as mean ± SD. **P*<0.05, ***P*<0.01.


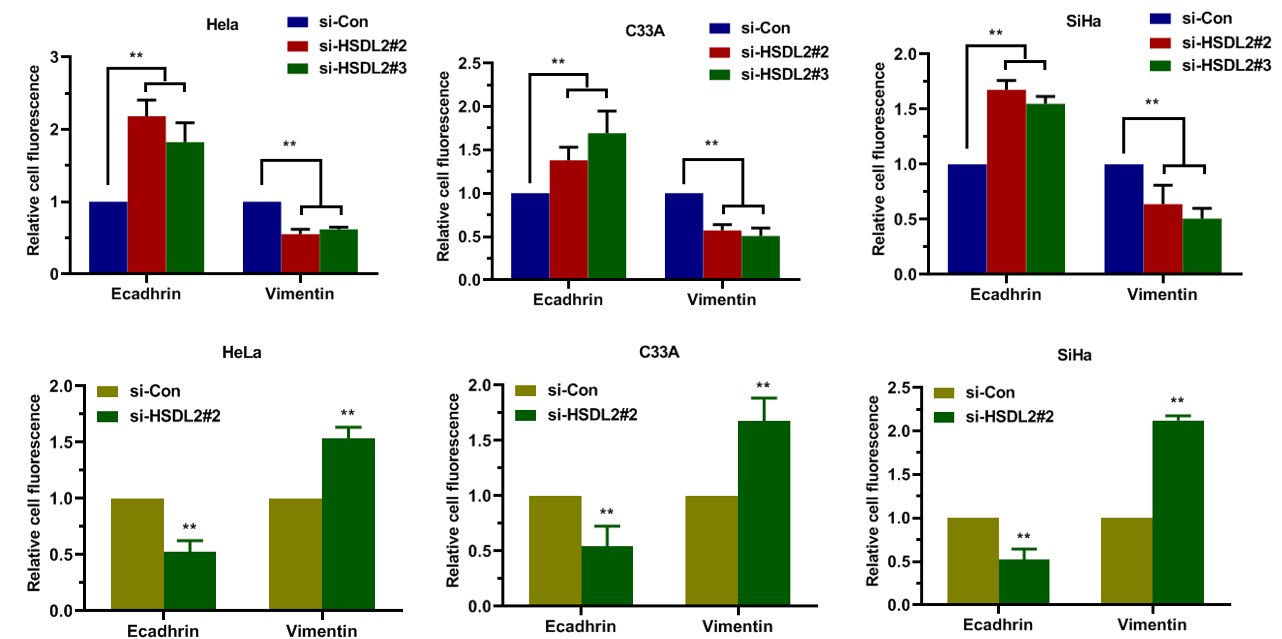


**Supplemental Figure 3:** Statistics of IF for Figure 5B. The data were presented as mean ± SD. **P*<0.05, ***P*<0.01.

**Supplementary Tables:**

**Supplementary Tables1. Abbreviations**

| WHO | world health organization |
| --- | --- |
| HPV | Human papillomavirus |
| LNMICC | lymph node metastasis of primary cervical cancer |
| CINs | cervical intraepithelial neoplasias |
| SPSS | Statistical Product and Service Solutions |
| IHC | Immunohistonchemistry |
| PBS | phosphate buffer saline |
| DMEM | dulbecco's modified eagle medium |
| DMSO | Dimethyl sulfoxide |
| MTT | 3-(4,5-dimethyl-2-thiazolyl)-2,5-diphenyl-2-H-tetrazolium bromide，Thiazolyl Blue Tetrazolium Bromide |
| TBST | Tris Buffered Saline Tween |
| SDS-PAGE | sodium dodecyl sulfate polyacrylamide gel electrophoresis |
| MMP-2 | matrix matalloproteinases 2 |
| FASN | fatty acid synthase |
| ACSL1 | Long-chain fatty acyl-CoA1 |
| SREBP1 | Sterol Regulatory Element-Binding Protein 1 |
| GAPDH | glyceraldehyde phosphate dehydrogenase |
| HRP | Horseradish peroxidase |
| ECL | Electrochemiluminescence |
| FBS | Fetal Bovine Serum |
| si-Con | The control group of the inhibition group |
| EMT | epithelial-mesenchymal transition |
| GEPIA | Gene Expression Profiling Interactive Analysis |
| TNM | Tumor Node Metastasis |
| FABP5 | fatty acid binding protein 5 |
| PRRX-1 | paired related homeobox 1 |
| GO | gene ontology |

**Supplementary Table 2. Instruments/equipment**

| **Instruments** | **Vendors** | **Numbers** |
| --- | --- | --- |
| Centrifuge | HITACHI, China | LX-200 |
| Fluorescence microscope | Olympus, Japan | IXT1 |
| Refrigerated centrifuge | TECAN, Britain | CT15RE |
| Cell Incubator | Thermo, USA | 0816-2014 |
| ELIASA | TECAN, Britain | Infinite M200PRO |
| Gel Imager System | Bio-Rad | ChemiDoc^TM^ Touch |
